# Microbiota Analysis and Microbiological Hazard Assessment in Chinese Chive (*Allium tuberosum* Rottler) Depending on Retail Types

**DOI:** 10.4014/jmb.2112.12013

**Published:** 2021-12-23

**Authors:** Dong Woo Seo, Su-jin Yum, Heoun Reoul Lee, Seung Min Kim, Hee Gon Jeong

**Affiliations:** Department of Food Science and Technology, College of Agriculture and Life Sciences, Chungnam National University, Daejeon 305-764, Republic of Korea

**Keywords:** Microbiota, Chinese chive, food safety, Food-borne pathogen, 16S rRNA gene sequencing

## Abstract

Chinese chive (*Allium tuberosum* Rottler) has potential risks associated with pathogenic bacterial contamination as it is usually consumed raw. In this study, we investigated the microbiota of Chinese chives purchased from traditional markets and grocery stores in March (Spring) and June (Summer) 2017. Differences in bacterial diversity were observed, and the microbial composition varied across sampling times and sites. In June, potential pathogenic genera, such as *Escherichia*, *Enterobacter*, and *Pantoea*, accounted for a high proportion of the microbiota in samples purchased from the traditional market. A large number of pathogenic bacteria (*Acinetobacter lwoffii*, *Bacillus cereus*, *Klebsiella pneumoniae*, and *Serratia marcescens*) were detected in the June samples at a relatively high rate. In addition, the influence of the washing treatment on Chinese chive microbiota was analyzed. After storage at 26°C, the washing treatment accelerated the growth of enterohemorrhagic *Escherichia coli* (EHEC) because it caused dynamic shifts in Chinese chive indigenous microbiota. These results expand our knowledge of the microbiota in Chinese chives and provide data for the prediction and prevention of food-borne illnesses.

## Introduction

Food-borne illnesses are considered a major concern globally. The World Health Organization (WHO) has reported approximately 600 million food-borne illnesses and 420,000 deaths due to 31 global hazards (major pathogens) [1, 2]. In particular, numerous food-borne illnesses caused by fresh produce consumption, mainly raw vegetables and fruits contaminated by pathogenic bacteria, have been reported. *Escherichia coli* O104:H4, detected in sprouts, infected about 4,000 people across Europe (mainly Germany) in 2011 [3, 4]. *Salmonella*, found in cucumber, infected 907 people in the United States [5], while 205 people were infected with *E. coli* O157:H7 detected in spinach [6]. Hence, fresh produce has a high potential risk of causing food-borne illness outbreaks, as also shown by a previous reports [7, 8].

Fresh produce can be contaminated with pathogens through various sources, such as agricultural water, soil amendments, harvesting equipment, field workers, and retail handling [9]. As it is often consumed raw because of changes in taste and appearance, tissue damage, and destruction of nutrients, the potential risk of food-borne illness is increases. Although deactivation strategies, such as thermal sterilization are effective in suppressing pathogens [10], further studies are needed to identify ways to prevent food-borne illness and improve the safety of fresh produce.

As over 95% of the microbes in nature are currently difficult to cultivate, the limitations of conventional culture-based methods are clear [11, 12]. Recently, to overcome these limitations, researchers have analyzed all microbes in the community using metagenomic approaches and non-culture-based methods to analyze changes in microbial communities [13-15]. In particular, using phylogenetic marker gene (16S rRNA gene) sequencing to investigate the diversity and composition of microbial communities in a specific environment, such as fresh produce, can elucidate the relationship between food and indigenous microbiota, including the potential risk of food-borne illnesses [13-17].

Chinese chive (*Allium tuberosum* Rottler), mainly cultivated and consumed in Asian countries, is a vegetable belonging to the same family as garlic, onion, and leek [18]. It is a rich source of vitamins, fiber, and minerals [19]. It has been reported that *Allium* vegetables, including Chinese chive, contain sulfur-containing compounds that improve human conditions such as cardiovascular and inflammatory diseases [18]. However, the risk of food-borne illness due to consumption of Chinese chives is expected to be high because it is usually consumed raw. Vegetables, including Chinese chives, are usually purchased at traditional markets or grocery stores. Unlike grocery stores, which take appropriate measures, such as refrigeration for food safety, traditional markets are usually located outdoors and are equipped with minimal facilities [20]. In addition, vendor retail behavior has been reported to be associated with a relatively high risk of potential food-borne illnesses [21]. Therefore, it is essential to study the microbiological hazards in vegetables of each retail type (traditional market and grocery store) to prevent outbreaks of food-borne illness.

Although some studies on plant microbiota have been carried out to date, little is known about the bacterial communities found in Chinese chives. In this study, we investigated the retail type-related characteristics of the microbial community, including potential food-borne pathogens, in two groups of Chinese chives, harvested in March and June. Chinese chive samples were purchased from a traditional market and grocery store located in South Korea in March and June, and their microbiota were analyzed using 16S rRNA gene sequencing. Several well-known and multidrug-resistant bacteria were also quantified by quantitative real-time polymerase chain reaction (qRT-PCR) with specific primers. We investigated the influence of different storage temperatures on Chinese chive microbiota composition and food-borne pathogen (*E. coli* O157:H7) contamination. These results provide insight into the microbial communities in Chinese chives and call for more comprehensive studies to better determine the potential of food-borne illnesses caused by Chinese chive consumption.

## Materials and Methods

### Sample Preparation

A total of 80 Chinese chive (*Allium tuberosum* Rottler) samples in the same cultivar were purchased in March (April) and June (Summer) 2017 (maximum production times) from the traditional market and grocery store located in Seoul and Busan, the top two populated cities in South Korea (n = 20 per each sampling group). The average temperatures in March and June were 9.02 ± 4.30°C (max. 14.02 ± 4.75°C, min. 4.90 ± 4.26°C) and 22.36 ± 1.83°C (max. 26.87 ± 2.75°C, min. 18.88 ± 1.71°C), respectively. Chinese chive samples purchased from traditional markets were kept at room temperature, while samples from grocery stores were stored in a refrigerator (4.5 ± 0.3°C). Samples were randomly selected from each traditional market and grocery store and were immediately transferred to the laboratory at 4°C. Chinese chive (25 g) was mixed with 225 ml of buffered peptone water (Oxoid, UK) in a filter bag (FILTRA-BAG; Labplas, Canada). A homogenization process was conducted to detach bacterial cells from the Chinese chive using Bagmixer 400 (Interscience, France). The mixture was filtered through a filter bag and centrifuged at 3,134 ×*g* for 10 min at 4°C. After the supernatant was removed, the residue pellet was suspended in TES buffer (0.1 M NaCl, 10 mM Tris-HCl, 1 mM EDTA, pH 8.0) to eliminate impurities. The pellet was stored at −80°C prior to metagenomic DNA extraction.

### Metagenomic DNA Extraction

Metagenomic DNA was extracted using the modified phenol-chloroform method described in previous studies [22]. The pellets were suspended in 500 μl cetyltrimethylammonium bromide (CTAB; Daejung, Korea) buffer containing 1% polyvinylpyrrolidone (PVP; Sigma-Aldrich, USA) and 50 μl lysozyme solution (100 mg/ml, Biosesang, South Korea) to remove polyphenols. Pellet mixtures were incubated at 37°C for 1 h and frozen at -80°C for 10 min. The pellet mixtures were incubated at 37°C for 10 min for thawing. A total of 200 μl proteinase K mixture (140 μl 0.5 M EDTA, 20 μl proteinase K (20 mg/ml), and 40 μl 10% sodium dodecyl sulfate) was added to the pellet mixtures and incubated at 56°C for 1 h. Pellet mixtures were centrifuged at 21,206 ×*g* for 10 min, and the supernatant was transferred to a new tube. After 100 μl of 5 M NaCl and 80 μl CTAB/NaCl solution (NaCl 41 mg/ml and CTAB 100 mg/ml) were added, the supernatant was incubated at 65°C for 10 min. Then, the supernatant was mixed with the same volume of phenol/chloroform/isoamyl alcohol (25:24:1 (v/v/v), Sigma-Aldrich) and centrifuged at 21,206 ×*g* for 5 min. After the upper phase (aqueous phase) was transferred to a new microtube, it was mixed with an equal volume of chloroform and centrifuged at 21,206 ×*g* for 5 min. The upper phase was, then, transferred to a new tube. A total of 3 μl of RNase A (100 mg/ml, Qiagen, Germany) was added, and the mixture was incubated at 37°C for 10 min. Next, a 10% volume of 3 M sodium acetate (pH 5.0, SAMCHUN, South Korea) and two volumes ice-cold anhydrous ethanol were added to the DNA mixture, which was subsequently centrifuged at 21,206 ×*g* for 20 min at 4°C. After the supernatant was removed, the pellet was centrifuged at 21,206 ×*g* for 5 min at 4°C for washing. The pellet was dried at room temperature after the supernatant was discarded, and then, mixed with TE buffer and incubated at 55°C for 1 h. Using Optizen Nano Q (Mecasys, Korea), the purity and concentration of metagenomic DNA were measured, and the DNA was kept at −20°C.

### MiSeq Sequencing and Microbial Community Assemblage

The analysis was conducted using primers 799F-mod6 (5′-CMGGATTAGATACCCKGGT-3′) and 1114R (5′-GGGTTGCGCTCGTTGC-3′), which amplify the V5-V6 region of the 16S rRNA gene segment. These primers were designed to minimize PCR amplification of chloroplast and mitochondrial DNA [23, 24]. PCR amplification was conducted using PrimeSTAR HS DNA polymerase (Takara, Japan) and the following PCR protocol: initial denaturation (98°C, 3 min), 30 cycles of denaturation (98°C, 10 sec), annealing (57°C, 15 sec), elongation (72°C, 30 sec), and final elongation (72°C, 3 min). The PCR product was purified using the MEGAquick-spin Plus kit (iNtRON, Korea). Index PCR was conducted using the Illumina Nextera XT index kit (Illumina, USA), and the library was purified using AMPure XP beads (Beckman Coulter, USA). The size and quality of the library were validated using an Agilent Bioanalyzer 1000 chip (Agilent, USA) and the KAPA qPCR kit (KAPA Biosystems, USA). Paired-end (2 × 300 bp) sequencing analysis was performed based on the Illumina MiSeq platform (Illumina). Barcode sequences, primer sequences, low average quality score sequences (< 25), and homopolymers (> 8) in raw sequences were trimmed off using the MOTHUR software (ver. 1.38.1). The UCHIME algorithm in the MOTHUR software was used to eliminate chimeric sequences [25]. Determination of operational taxonomic units (OTUs), diversity analysis, and principal coordinate analysis (PCoA) were performed using the CLC Genomics Workbench (ver. 9.5.3, CLC bio, Denmark). Taxonomic assignment was determined using the SILVA database (ver. 123) with an 80% confidence threshold and clustered on the basis of 99% sequence similarity. Diversity comparison among samples was conducted using the α-diversity index (observed OTUs, Chao 1 index, and Shannon index) and validated reads (20,000 bp). β-diversity was illustrated through PCoA based on the Bray-Curtis distance. Heat map analysis of microbial abundance at the genus level was performed using the ‘heatmap.plus’ R package in RStudio (ver. 1.1.463).

### Bacterial Quantification Using qRT-PCR

To quantify the colony-forming units (CFUs) of total and pathogenic bacteria, primers for the 16S rRNA gene and specific virulence genes were used (Table S1). The qRT-PCR reaction was conducted using the CFX Connect Optics Module (Bio-Rad, USA) and KOD SYBR qPCR Mix (Toyobo, Japan). The PCR mixture (20 μl) consisted of 10 μl of KOD SYBR qPCR Mix, 7 μl of distilled water, 1 μl of 10 μM forward primer, 1 μl of 10 μM reverse primer, and 1 μl of DNA template. Standard curves for quantification were generated using the log-concentration of serial dilutions [16, 17, 26]. Bacterial loads were calculated by comparing Ct values with the standard curve. The regression coefficients (r^2^) of all the standard curves were higher than 0.99.

### Experimental Enterohemorrhagic *E. coli* Infection Model

Enterohaemorrhagic *E. coli* (EHEC) O157:H7 ATCC 35150 was cultured at 37°C in Luria-Bertani (LB) medium, and the number of infected cells was 10^5^ CFU per an each sample. Considering the purchase type of Chinese chive, the model experiment was conducted at various storage conditions including washing, unwashing, and two temperatures (26°C and 4°C) in triplicate. Unwashed Chinese chives were purchased from a traditional market in June. Before EHEC infection, samples were washed using the method suggested by the Ministry of Food and Drug Safety (South Korea). After dipping the Chinese chive in water for 1 min, the process of washing in new water for 30 s was repeated twice, and then dried. EHEC was evenly spotted (10 μl) on the surface of washed and unwashed samples and stored at 26°C and 4°C for 18 h. An uninfected group was also used as a control at the same temperatures and for the same time periods. Bacterial cells on Chinese chive were peeled off as described in the sample preparation section at each time point (0, 6, and 12 h), and these were stored at −80°C until metagenomic DNA extraction. To determine the difference in microbiota composition between groups, linear discriminant analysis effect size (LEfSe) was conducted using the online LEfSe tool via the Huttenhower Lab Galaxy server (https://huttenhower.sph.harvard.edu/galaxy).

### Statistical Analysis

Statistically significant differences between groups were verified using the Student’s *t*-test or Duncan’s multiple range test after the Shapiro-Wilk normality test to analyze all data except network analysis data (correlation coefficient). α-diversity and PCoA were computed and evaluated for microbial communities using the CLC Microbial Genomics Module at the OTU level. Statistical analysis was conducted using SAS (ver. 9.4) and PRISM (ver. 5.02). Differentially abundant genera that were statistically significant using an alpha level of 0.05, and exceeded an LDA log score of ± 2.0, in LEfSe, were included in the bar plot.

## Results and Discussion

### Differences of Diversity Indices and Bacterial Amounts

A total of 4,789,234 reads (average 59,865 reads) from 80 Chinese chive samples were analyzed to examine the bacterial community. The analyzed reads were randomly normalized to 20,000 reads per sample to compare diversity indices among Chinese chive samples. The diversity indices and bacterial cell number differed significantly depending on the sampling time and site (Table 1). When the number of microbes was compared according to the sampling time, the total bacterial number in the June sample (7.41 × 10^6^ CFU/g) was higher than that in the March sample (3.46 × 10^5^ CFU/g) (*p* < 0.0001). All the diversity indices of the March samples, including OTUs (431.05 ± 122.22 in March and 275.45 ± 95.99 in June), Chao 1 (621.49 ± 152.56 in March and 447.58 ± 154.11 in June), and Shannon diversity (4.95 ± 0.81 in March and 3.85 ± 0.79 in June), were statistically higher than those of the June samples (*p* < 0.0001). However, no significant differences were observed between the sampling locations (Seoul and Busan), consistent with the results of a previous report showing the bacterial diversity and population characteristics in lettuce and perilla leaves [16, 17, 27]. It is well known that the diversity and population of bacteria in plants can generally be influenced by environmental factors, such as temperature, humidity, soil conditions, and interactions among microbes [28, 29]. When all samples were collected in this study, the temperature in June (22.36 ± 1.83°C) was relatively higher than that in March (9.02 ± 4.30°C). Thus, the temperature difference was expected to strongly affect the bacterial diversity and population in our samples.

The effects of other parameters, retail types, were also apparent. When we collected the samples, Chinese chive were displayed on stalls without any preservation control at traditional markets, while these were refrigerated in a container or sealed plastic bag at grocery stores. Therefore, our results showing the differences of bacterial diversity and population seemed to be attributed to retail types.

### Cluster Analysis of Microbiota Composition

**Fig. 1:**
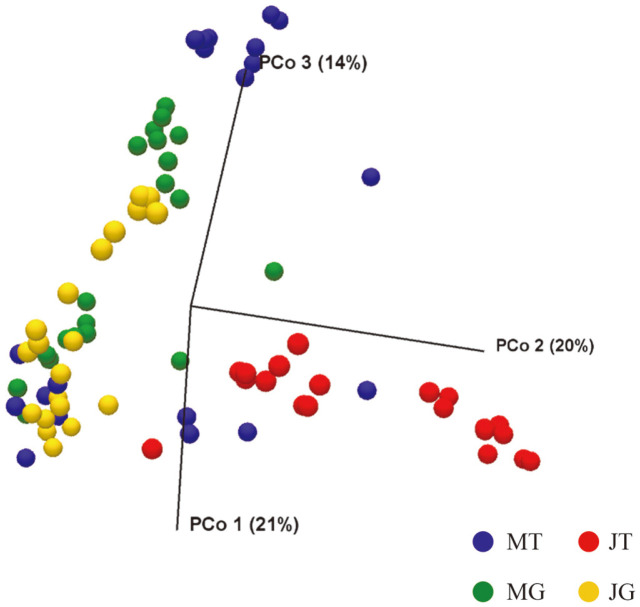
Principal coordinates analysis plot of Bray-Curtis distance among Chinese chive samples. Each group is represented as color. The percentage contributions to the variance of the data from principal components 1, 2 and 3 (PCo 1, PCo 2, and PCo 3) are listed along axes representing them. (MT: March-traditional market, JT: June-traditional market, MG: March-grocery store, and JG: June-grocery store).

We analyzed the microbiota using PCoA based on Bray-Curtis distance with permutational multivariate analysis of variance (PERMANOVA), which demonstrated clustering by sampling time-retail type (*p* < 0.0001). As shown in Fig. 1, June-traditional market (JT) and June-grocery store (JG) samples formed separate clusters on the PCoA plot. In particular, the JT samples were clearly separated from the rest of the samples. The average temperature in June was higher than that in March. In addition, vendor behavior, including refrigeration conditions in the traditional market, was not appropriate. In previous studies elucidating the microbiota of raw vegetables, distinct clustering patterns were observed in the unweighted and weighted matrices according to sampling times, and this appears to have been attributed to the difference in temperature [16, 27]. Chinese chives were transferred to each retail type and stored for 28–32 h until they were purchased by customers. Thus, these results may explain why the structure of the bacterial community in Chinese chives was strongly affected by retail type and high temperature, consistent with the results of α-diversity analysis.

### Microbial Signatures at the Phylum and Class Levels

**Fig. 2:**
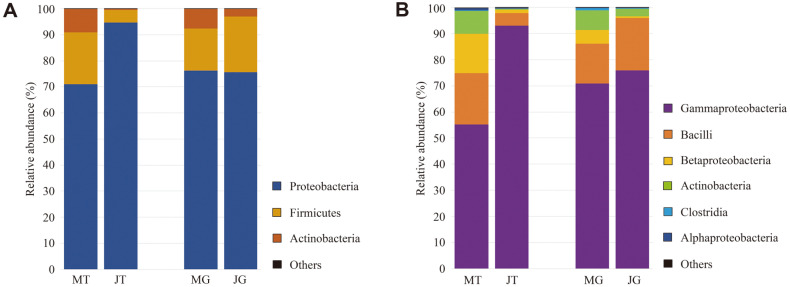
Comparison of microbiota composition of the Chinese chive. At the (**A**) phylum and (**B**) class levels. ‘Others’ indicate microbial phyla/classes with relative abundance below 1% in at least one sample, respectively. (MT: March-traditional market, JT: June-traditional market, MG: March-grocery store, and JG: June-grocery store).

The difference in the bacterial composition of Chinese chive according to sampling time and retail type was analyzed taxonomically at each level. At the phylum level, Proteobacteria, Firmicutes, and Actinobacteria were mainly found in all samples (Fig. 2A). High numbers of Proteobacteria were observed in the JT group (94.60 ± 7.29%, *p* < 0.001) compared to those of other groups (71.20 ± 25.64% in March-traditional market (MT), 76.00 ± 17.08% in March-grocery store (MG), and 75.70 ± 16.33% in JG), whereas the numbers of Firmicutes were the lowest in the same group (4.93 ± 7.14%, *p* < 0.05). Acinetobacter was mostly found in MT samples (8.90 ± 5.65 %, *p* < 0.001), and its prevalence was significantly different between the JT (0.49 ± 0.41%) and JG (3.10 ± 2.29%) groups.

At the class level, Gammaproteobacteria, Bacilli, and Betaproteobacteria were the main components in all samples (Fig. 2B). The relative abundance of Gammaproteobacteria belonging to Proteobacteria was the highest in the JT group (92.90 ± 8.08 %, *p* < 0.001) compared to that in the other groups (55.30% ± 18.64% in MT, 70.65%± 12.86% in MG, 74.55% ± 16.65% in JG). In contrast, the number of Bacilli belonging to Firmicutes was the lowest in the JT group (4.92 ± 7.14 %, *p* < 0.05), while no statistically significant differences in these bacterial numbers were observed among the other groups (*p* > 0.46). In addition, higher numbers of Betaproteobacteria were observed in the MT group (15.15 ± 15.85%, *p* < 0.05) compared to those in the other groups (5.12 ± 5.81% in MG, 1.21 ± 1.48% in JT, 0.68 ± 0.66% in JG). This is consistent with the results of previous studies showing the relative abundance of dominant phyla and classes in the plant phyllosphere [16, 17, 29-31].

### Comparison of Microbiota Composition according to Sampling Time and Retail Type at the Genus Level

**Fig. 3:**
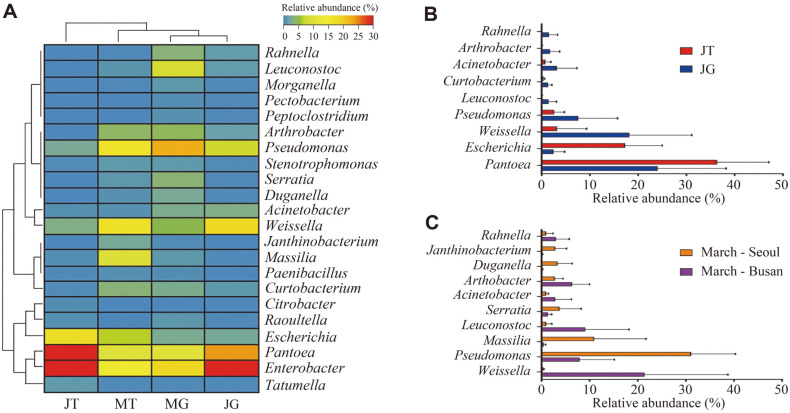
Analysis of microbiota composition at the genus level on Chinse chive. (**A**) Heat-map analysis shows the genus level relative abundance (more than average 0.5% at each group) on Chinse chive samples. Samples were clustered by Spearman’s rank correlation. (MT: March-traditional market, MG: March-grocery store, JT: June-traditional market, JG: Junegrocery store). (**B**) The relative abundance of genera in Chinese chive samples with statistically significant differences between June-traditional market (JT) and June-grocery store (JG). (**C**) The relative abundance of genera with statistical differences between March-Seoul and March-Busan.

We analyzed the microbiota at the genus level and visualized the differences among each group (MT, MG, JT, and JG groups) using a heat map (over an average of 0.5% in each group) (Fig. 3A). Differences in the proportions of some genera were observed among the groups. In the June groups, *Enterobacter* was found at a higher percentage than that in the March groups (averages of 16.48 ± 6.71% and 33.65 ± 12.88% in the March and June groups, respectively, *p* < 0.0001). A higher proportion of *Enterobacter* was also found in lettuce cultivated at hot temperatures compared to that in lettuce cultivated at low temperatures [16]. *Enterobacter* is recognized as an important pathogen in relation to disease and antibiotic resistance. *E. sakazakii*, *E. agglomerans*, *E. aerogenes*, and *E. cloacae* are representative pathogens that cause endocarditis, urinary tract infections, septic arthritis, and osteomyelitis [32]. *Pantoea* was also detected at a higher proportion in the June group than in the March group (averages of 10.58% ± 4.31% and 30.10 ± 14.09% in the March and June groups, respectively, *p* < 0.0001). *Pantoea* is reported to grow most effectively at 28–30°C, which is relatively similar to the temperature in June [33, 34]. In the March group, the relative abundance of *Pseudomonas* was higher than that in the June samples (averages of 19.41 ± 14.29% and 4.98 ± 6.55% in the March and June groups, respectively, *p* < 0.0001). Some *Pseudomonas* species have been reported to be able to survive at low temperatures, and physiological changes that neutralize problems caused by low temperatures have been observed in these species [35, 36]. Certain *Pseudomonas* species, such as *P. aeruginosa*, known to be pathogenic, require attention [37]. The proportion of *Arthrobacter* in the March group was also higher than that of June samples (averages of 4.45 ± 3.47% and 0.86 ± 1.68% in the March and June groups, respectively, *p* < 0.0001). *Arthrobacter* is frequently found in the soil and rhizosphere of various plants; some *Arthrobacter* species, such as *A. nitroguajacolicus* and *A. agilis*, promote plant growth [38-40]. The pigments produced by *Arthrobacter* are related to the stabilization of the cell membrane at low temperatures [41].

Differences between traditional markets and grocery stores were observed in the relative abundance of some genera in samples purchased in June (over an average of 1% in each group) (Fig. 3B). In the JT group, the relative abundance of *Pantoea* was higher than that in the JG group (*p* < 0.01). The JT group was likely to be exposed to a relatively high temperature in June during the retail process. Some *Pantoea* species, such as *P. agglomerans*, are known to cause soft tissue, bone, joint, and bloodstream infections [42]. *Escherichia* was also found at a higher percentage in the JT group (*p* < 0.0001). Because *Escherichia* is frequently found on human skin, vegetables can be contaminated with *Escherichia* during retail handling. It is assumed that *E. coli* can multiply in chives during the retail process because vegetables in the market are usually kept at room temperature [20, 21]. Some *Escherichia* strains, such as EHEC or enterotoxigenic *E. coli* (ETEC) are pathogenic and cause a large number of infections worldwide [43]. These results suggest that Chinese chives purchased in traditional markets may have a relatively high contamination risk for some potential genera.

In the March groups, the relative abundance of some genera differed depending on the sampling region (Fig. 3C). *Pseudomonas*, *Massilia*, *Serratia*, *Dnganella*, and *Janthinobacterium* were present in higher proportions in the March-Seoul group compared to the March-Busan group. In contrast, the relative abundance of *Weissella*, *Leuconostoc*, *Arthrobacter*, and *Rahnella* was higher in the March-Busan group. *Pseudomonas*, *Massilia*, *Serratia*, *Dnganella*, *Janthinobacterium*, *Arthrobacter*, and *Rahnella* are commonly found in soil, plant surfaces, and water [44-50]. However, *Weissella* and *Leuconostoc*, classified as lactic acid bacteria, are associated with fermented vegetables [51]. These seemed to reflect regional characteristics, including distribution conditions, in the March groups.

### Quantification of Potential Pathogens in Chinese chive (log CFU/g of each sample)

We detected and quantified pathogens (EHEC, Enteropathogenic *E. coli*, *Staphylococcus aureus*, *Acinetobacter lwoffii*, *Bacillus cereus*, *Klebsiella pneumoniae*, and *Serratia marcescens*) in Chinese chive using qRT-PCR (Table 2). Those pathogenic species were selected based on the relative abundances of potential pathogenic genera among various food-borne pathogens frequently reported.

*Acinetobacter lwoffii* was detected in the MT (6.33 × 10^3^ – 8.19 × 10^3^ CFU/g, n = 2) and JG groups (6.20 × 10^4^ –4.37 × 10^5^ CFU/g, n = 10), and that was detected in half and the highest value was measured in the JG group. *A. lwoffii* is a potential opportunistic pathogen that has been reported to cause sepsis, pneumonia, and meningitis [52]. *B. cereus*, which has various virulence factors, such as enterotoxin, emetic toxin and hemolysin, and can resist heat treatment and gamma-ray irradiation through spore formation, was only detected in the JT group (2.71 × 10^3^ – 7.52 × 10^3^ CFU/g) [53]. *K. pneumoniae* was only detected in June samples (n = 17), with a higher value and detection rate in the JT group (1.91 × 10^1^ – 5.78 × 10^4^ CFU/g) compared to that in the JG group (4.33 × 10^1^ – 1.78 × 10^3^ CFU/g). *K. pneumoniae* is an opportunistic pathogen that accounts for 10% of nosocomial bacterial infections and causes kidney failure, lung infections, and encephalitis [54, 55]. EHEC produces Shiga toxins, that are associated with the development of severe complications of infection such as hemorrhagic colitis and hemolytic uremic syndrome [43]. Enteropathogenic *E. coli* (EPEC) remains an important cause of fatal infant diarrhea in developing countries, however the mechanism that causes diarrhea is unknown [43]. *S. aureus* is one of the widespread food-borne pathogens, that produces many virulence factors, including immune-modulatory factors, toxins, and exoenzymes [56]. EHEC, EPEC, and *S. aureus* were not detected in any of the samples. Overall, the bacterial amounts and detection rate of pathogens in the June groups were higher than those in the March groups. The relatively higher temperature in June compared to that in March likely affected pathogen growth. These results show that Chinese chives in June may have a higher risk of food-borne illness than those in March.

### Effect of Enterohemorrhagic *E. coli* (EHEC) Infection on Chinese chive Indigenous Microbiota over Time

Although EHEC was not detected in this study, these pathogenic strains are frequently associated with the consumption of EHEC-contaminated fresh produce [57, 58]. In addition, since many households are washing vegetables to remove impurities before consumption, the washing process was considered an important factor and was included in this experiment.

**Fig. 4:**
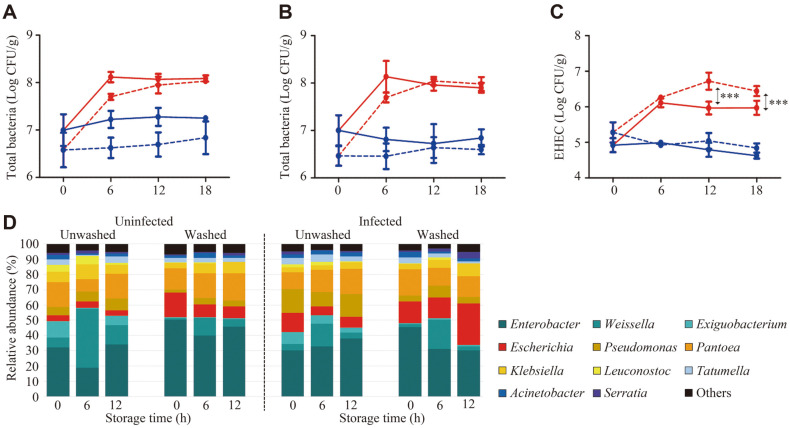
Change in pathogen (EHEC) and microbiota on Chinese chive depending on storage temperature and washing condition. The number of total bacteria and EHEC were quantified in Chinese chive stored at 4°C (filled blue circle) or 26°C (filled red circle) after unwashed (solid line) or washed (dotted line). Amounts of total bacterial loads in (**A**) uninfected and (**B**) infected groups over time. (**C**) Bacterial loads of EHEC in the EHEC-infected Chinese chive over time. (***, *p* < 0.001). (**D**) Shifts in microbiota composition at the genus level of Chinese chive samples following experimental contamination with EHEC and storage under different washing conditions at 26°C.

We obtained a total of 2,615,320 reads (average 72,648, *n* = 36) to analyze the microbiota of the experimental samples. Changes in the number of total bacteria and EHEC due to various factors (washing treatment, EHEC infection, storage temperature, and time) were observed using qRT-PCR (Figs. 4A–4C). The total bacterial and EHEC numbers were not significantly altered at 4°C regardless of the washing treatment, EHEC infection, or storage time. In contrast, the total bacterial load of Chinese chive increased until 6 h of storage at 26°C. Generally, it is well known that the bacterial population more significantly increased during fresh-cut vegetable storage at a higher temperature [59]. Compared with the unwashed Chinese chive, the EHEC number in the washed groups was statistically higher at 12 h and 18 h during storage at 26°C. At 12 h, the average number of EHEC was 5.96 ± 0.18 log CFU/g in the unwashed groups, but 6.72 ± 0.24 log CFU/g in the washed groups (*p* < 0.001). These features were observed after 18 h. The average numbers of EHEC in the unwashed and washed groups were 5.97 ± 0.20 log CFU/g and 6.44 ± 0.14 log CFU/g, respectively (*p* < 0.001).

A shift of indigenous microbiota according to experimental conditions was also observed using 16S rRNA gene-based sequencing (Fig. 4D). Sequencing analysis was conducted only using samples incubated at 26°C. The relative abundance of *Escherichia* in infected and washed samples increased after 12 h (average from 14.00 ± 5.57%to 27.33 ± 1.53%, *p* < 0.05), and it was the highest among all samples. Given that a change in the relative abundance of *E. coli* was only observed in the infected and washed samples, EHEC infection is expected to account for a large portion of *Escherichia*-caused infections. It is well known that the optimal growth temperature of the *Escherichia* genus is 37°C in various culture environments. Therefore, the increased relative abundance of *Escherichia* was associated with a higher storage temperature. As the proportion of *Escherichia* in infected and washed samples increased, the proportion of *Serratia* (average from 0.87 ± 0.23% to 4.00% ± 1.00%, *p* < 0.01) and *Klebsiella* (average from 3.67 ± 1.15% to 8.33 ± 1.53%, *p* < 0.05) in infected and washed samples also increased after 12 h. In contrast, the relative abundance of *Enterobacter* in infected and washed samples decreased after 12 h (average from 45.00 ± 2.00% to 30.33 ± 2.31%, *p* < 0.01).

**Fig. 5:**
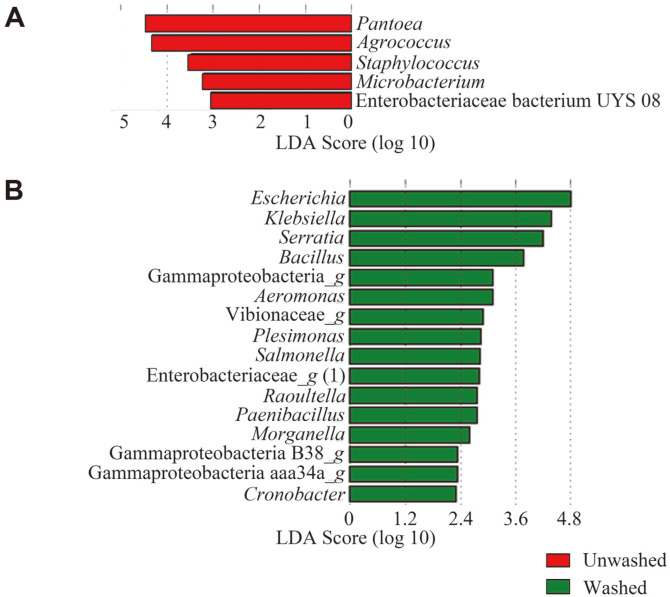
Linear discriminant analysis effect size (LEfSe) comparing differences in abundant genera on Chinese chives infected with EHEC according to washing process at 12 h storage. Shifts in abundant genera in (**A**) unwashed (red) and (**B**) washed Chinese (green) chives. After 12 h of storage compared to 0 h, the bacteria with statistically significant change (LDA score ≥ 2, *p* < 0.05) in the relative abundance is shown alongside the horizontal lines.

The effect of EHEC infection in unwashed and washed samples on the shift in indigenous microbiota was also analyzed using LEfSe (Fig. 5). An obvious variety of microbiota shifts were observed in the washed groups compared to the unwashed groups infected with EHEC during storage (26°C for 12 h). Only five genera, *Pantoea*, *Agrococcus*, *Staphylococcus*, *Microbacterium*, and *Enterobacter*iaceae bacterium UYS 08, were significantly increased in the unwashed groups infected with EHEC (LDA score ≥ 2.0; *p* < 0.05). In contrast, 36 genera were shifted in the washed groups infected with EHEC. Among these genera, potential pathogens, such as *Escherichia*, *Klebsiella*, *Serratia*, *Bacillus*, and *Salmonella* were significantly increased (LDA score ≥ 2.0; *p* < 0.05). These results indicate that some bacteria, including pathogens, on Chinese chive multiply better at 26°C, and are more likely to increase the risk of food-borne illness when stored after washing. These results also suggested that the washing process removed some of the resident microbes, and as a result artificially infected EHEC colonized better and affected indigenous microbiota more. In addition, the microbiota of Chinese chive is strongly related to the storage conditions (temperature and washing) and may influence the outbreak of food-borne illnesses. Therefore, further studies on the pathogenicity of Chinese chives are needed to analyze in detail the risk factors involved and improve the safety of fresh produce such as ready-to-eat salad.

## Supplemental Materials

Supplementary data for this paper are available on-line only at http://jmb.or.kr.

## Figures and Tables

**Table 1:** Summary of α-diversity indices obtain from 16S rRNA gene sequencing and total bacterial loads in the Chinese chive at each sampling time and retail.

Sampling time	Retail type	Average reads	Normalized reads	Observed OTUs	Chao 1	Shannon	CFU/g
March	Traditional market (*n*=20)	40,829	20,000	472.23 ^A^	647.86 ^A^	4.83 ^A^	9.24 × 10^4 A^
	Grocery store (*n*=20)	53,404		389.88 ^B^	595.13 ^A^	5.07 ^A^	6.00 × 10^5 B^
June	Traditional market (*n*=20)	69,852		283.02 ^C^	460.16 ^B^	4.05 ^B^	8.93 × 10^6 C^
	Grocery store (*n*=20)	75,377		267.87 ^C^	435.00 ^B^	3.64 ^B^	5.90 × 10^6 C^

^A-C^Means with different letters are significantly different at *p* < 0.05 (Duncan's multiple range test)

**Table 2:** Quantification of pathogenic bacteria through quantitative real time polymerase chain reaction (qRT-PCR).

Pathogenic bacteria	Sampling time	Retail type	Bacterial load (CFU/g)	Detection rate (*n* = 20)
*Acinetobacter lwoffii*	March	Traditional market	7.26 × 10^3^	10% (2)
		Grocery store	N.D.	0%
	June	Traditional market	N.D.	0%
		Grocery store	1.56 × 10^5^	50% (10)
*Bacillus cereus*	March	Traditional market	N.D.	0%
		Grocery store	N.D.	0%
	June	Traditional market	5.73 × 10^3^	15% (3)
		Grocery store	N.D.	0%
*Klebsiella pneumoniae*	March	Traditional market	N.D.	0%
		Grocery store	N.D.	0%
	June	Traditional market	5.18 × 10^3^	60% (12)
		Grocery store	5.03 × 10^2^	25% (5)
*Serratia marcescens*	March	Traditional market	7.34 × 10^2^	35% (7)
		Grocery store	N.D.	0%
	June	Traditional market	N.D.	0%
		Grocery store	2.89 × 10^3^	50% (10)
Enterohemorrhagic *Esherichia coli*(EHEC)	March	Traditional market	N.D.	0%
		Grocery store	N.D.	0%
	June	Traditional market	N.D.	0%
		Grocery store	N.D.	0%
Enteropahtogenic *Esherichia coli*(EPEC)	March	Traditional market	N.D.	0%
		Grocery store	N.D.	0%
	June	Traditional market	N.D.	0%
		Grocery store	N.D.	0%
Enterotoxigenic *Esherichia coli*(ETEC)	March	Traditional market	N.D.	0%
		Grocery store	N.D.	0%
	June	Traditional market	N.D.	0%
		Grocery store	N.D.	0%
*Staphylococcus aureus*	March	Traditional market	N.D.	0%
		Grocery store	N.D.	0%
	June	Traditional market	N.D.	0%
		Grocery store	N.D.	0%

(N.D., non-detected).
