# Supplementary material for: Microbiota Analysis and Microbiological Hazard Assessment in Chinese Chive (Allium tuberosum Rottler) Depending on Retail Types
Source: J Microbiol Biotechnol. 2021 Dec 23;32(2):195–204. doi: 10.4014/jmb.2112.12013 (PMC9628847; doi:10.4014/jmb.2112.12013)
Supplement: Supplementary file 1 [file jmb-32-2-195-supple.pdf]

J. Microbiol. Biotechnol.
https://doi.org/10.4014/jmb.2112.12013

Supplement table 1. PCR primers information used in this study.

Target bacteria	Target gene	Primer sequence (5' → 3')	References
Acinetobacter lwoffii	*bla*$_{OXA-134-like}$	F : CAGGAAGTACAACGCATCCA<br>R : TGCTGGACTTGAGGATCAAA	[1]
Klebsiella pneumoniae	Internal transcribed spacer region	F : ATTTGAAGAGGTTGCAAACGAT<br>R : CCGAAGATGTTTCACTTCTGATT	[2]
Staphylococcus aureus	Sa442	F : GTCGGGTACACGATATTCTTCACG<br>R : CTCGTATGACCAGCTTCGGT	[3]
Serratia marcescens	*luxS*	F : TGCCTGGAAAGCGGCGATGG<br>R : CGCCAGCTCGTCGTTGTGGT	[4]
Enterohemorrhagic *Escherichia coli*	*stx1*	F : GTCACAGTAACAAACCGTAACA<br>R : TCGTTGACTACTTCTTATCTGGA	[5]
Enterotoxigenic *Esherichia coli*	*st*	F : TTTCCCCTCTTTTAGTCAGTCAA<br>R : GCAGGATTACAACACAATTCACAGCAG	[6]
Enteropahtogenic *Esherichia coli*	*eaeA*	F : ATGCTTAGTGCTGGTTTAGG<br>R : GCCTTCATCATTTCGCTTTC	[7]
Total bacteria	16s rRNA V5-V6	F : CMGGATTAGATACCCKGGT<br>R : GGGTTGCGCTCGTTGC	[8]

References

1. Kamolvit W, Higgins PG, Paterson DL, Seifert H. 2014. Multiplex PCR to detect the genes encoding naturally occurring oxacillinases in *Acinetobacter* spp. *J. Antimicrob. Chemother.* **69:** 959-963.
2. Liu Y, Liu C, Zheng W, Zhang X, Yu J, Gao Q*, et al.* 2008. PCR detection of *Klebsiella pneumoniae* in infant formula based on 16S–23S internal transcribed spacer. *Int. J. Food Microbiol.* **125:** 230-235.
3. Reischl U, Linde H-Jr, Metz M, Leppmeier B, Lehn N. 2000. Rapid identification of methicillin-resistant *Staphylococcus aureus* and simultaneous species confirmation using real-time fluorescence PCR. *J. Clin. Microbiol.* **38:** 2429-2433.
4. Salamon D, Gosiewski T, Krawczyk A, Sroka-Oleksiak A, Duplaga M, Fyderek K*, et al.* 2020. Quantitative changes in selected bacteria in the stool during the treatment of Crohn's disease. *Adv Med Sci.* **65:** 348-353.
5. Jothikumar N, Griffiths MW. 2002. Rapid detection of *Escherichia coli* O157: H7 with multiplex real-time PCR assays. *Appl. Environ. Microbiol.* **68:** 3169-3171.
6. Chukwu MO, Abia ALK, Ubomba-Jaswa E, Obi LC, Dewar JB. 2019. Antibiotic resistance profile and clonality of *E. coli* isolated from water and paediatric stool samples in the north-west, province South Africa. *J. Pure Appl. Microbiol.* **13:** 517-530.
7. Vahedi A, Soltan Dallal MM, Douraghi M, Nikkhahi F, Rajabi Z, Yousefi M*, et al.* 2018. Isolation and identification of specific bacteriophage against enteropathogenic *Escherichia coli* (EPEC) and in vitro and in vivo characterization of bacteriophage. *FEMS Microbiol. Lett.* **365:** fny136.
8. Yu Y-C, Yum S-J, Jeon D-Y, Jeong H-G. 2018. Analysis of the microbiota on lettuce (*Lactuca sativa* L.) cultivated in South Korea to identify foodborne pathogens. *J. Microbiol. Biotechnol.* **28:** 1318-1331.
